# Comprehensive analysis of metabolism-related genes in sepsis reveals metabolic–immune heterogeneity and highlights GYG1 as a potential therapeutic target

**DOI:** 10.3389/fimmu.2025.1682846

**Published:** 2025-11-17

**Authors:** Jie Zheng, Kangjie Qin, Xiaoqin Wang, Banghai Feng, Yuting Zhang, Yiyu Wang, Han Qin, Qiuyu Dai, Xinxin Liu, Kun Yu, Song Qin

**Affiliations:** 1 Department of Critical Care Medicine, Affiliated Hospital of Zunyi Medical University, Zunyi, Guizhou, China; 2 Department of Pediatrics, The Second Affiliated Hospital of Zunyi Medical University, Zunyi, Guizhou, China; 3 Department of Critical Care Medicine, Zunyi Hospital of Traditional Chinese Medicine, Zunyi, Guizhou, China; 4 Department of Respiratory and Critical Care Medicine, Kweichow Moutai Hospital, Renhuai, Guizhou, China

**Keywords:** sepsis, metabolism, immune infiltration, risk score, lipid nanoparticles, GYG1

## Abstract

**Background:**

Sepsis is a life-threatening syndrome characterized by dysregulated host immune responses, yet the metabolic drivers of immune dysfunction remain poorly understood.

**Methods:**

Here we systematically profiled metabolism-related genes (MRGs) in sepsis using bulk transcriptomic data and stratified patients into two subgroups with distinct immune infiltration profiles by MRGs, as assessed by CIBERSORT and single-cell RNA-seq integration. Machine learning identified five hub metabolic genes for constructing a metabolic risk score, whose prognostic relevance was robustly validated in an external cohort. Single cell analyses, cell–cell communication, and cell-type-specific differential expression analyses were performed to dissect the immunological context. Finally, *in vivo* validation was conducted using an LPS-induced sepsis mouse model.

**Results:**

Patients in the high metabolic risk group exhibited a neutrophil-dominant and lymphocyte-suppressed immune landscape, consistent across bulk and single-cell analyses. Among the five hub genes (ALPL, CYP1B1, GYG1, OLAH, VNN1), GYG1 demonstrated the strongest predictive performance and was highly expressed in monocytes, neutrophils, and proliferating myeloid cells. High-risk patients displayed intensified monocyte–dendritic cell interactions and transcriptional programs enriched in neutrophil degranulation pathways. *In vivo*, Gyg1 was markedly upregulated in septic mice, and LNP-mediated siRNA knockdown of Gyg1 significantly improved survival in the LPS model. Mechanistically, Gyg1 knockdown significantly reduced glycogen content in myeloid cells, attenuated IL-6 and TNF-α production, alleviated LPS-induced neutrophil, and modestly decreased CD40 expression in monocytes and dendritic cells. These results collectively suggest that Gyg1 regulates metabolic fueling of inflammatory activation and intercellular communication during sepsis.

**Conclusions:**

This integrative multi-omics study established a robust immune–metabolic risk score system to predict sepsis patient outcomes and identified GYG1 as a metabolic driver of innate immune hyperactivation. Targeting GYG1 via LNP–siRNA delivery reduces glycogen availability and inflammatory output in myeloid cells, mitigating immune overactivation and improving disease outcomes *in vivo*, thereby highlighting its potential as a novel therapeutic target for sepsis.

## Introduction

Sepsis is a life-threatening syndrome characterized by dysregulated host responses to infection, leading to life-threatening organ dysfunction and high mortality worldwide (1–3). Despite advances in supportive care, effective targeted therapies remain elusive, largely due to the substantial biological heterogeneity of the disease. This heterogeneity is driven by complex interactions between immune and metabolic pathways, which together determine the trajectory from infection to systemic inflammation, immune suppression, and multi-organ failure (4).

Metabolic reprogramming is now recognized as a hallmark of immune cell activation and function during sepsis (5–7). Innate immune cells, such as neutrophils and monocytes, rapidly shift their metabolic profiles to meet the energy demands of pathogen clearance, whereas adaptive immune cells undergo distinct metabolic adaptations that influence survival and effector function (8, 9). Disruption of these tightly regulated metabolic–immune interactions can exacerbate inflammation or promote immune paralysis, both of which contribute to poor clinical outcomes. However, the precise metabolic drivers of immune dysregulation in sepsis remain incompletely understood.

High-throughput transcriptomic profiling has enabled comprehensive interrogation of gene expression programs underlying sepsis pathophysiology (10–12). While previous studies have examined immune-related genes or signaling pathways, few have systematically explored metabolism-related genes (MRGs) in the context of sepsis, particularly in relation to immune cell composition, intercellular communication, and patient prognosis (13–15). Furthermore, integration of bulk transcriptomics with single-cell RNA sequencing (scRNA-seq) offers an unprecedented opportunity to link transcriptional alterations to specific immune cell populations and to identify cell-type-specific therapeutic targets.

In this study, we performed an integrative multi-omics analysis of MRGs in sepsis, combining bulk RNA-seq, external cohort validation, and single-cell transcriptomic datasets. We established a robust immune–metabolic risk score system that stratifies patients into distinct metabolic–immune subtypes with divergent immune infiltration patterns and predicted outcomes. Through machine learning–based feature selection, we identified five hub genes driving this stratification and validated their prognostic performance in an independent dataset. Among them, GYG1 emerged as a key gene associated with innate immune hyperactivation, predominantly expressed in neutrophils and monocytes. Functional validation in an LPS-induced sepsis mouse model demonstrated that lipid nanoparticle (LNP)–mediated siRNA silencing of Gyg1 significantly ameliorated disease severity. Our findings provide new insights into the metabolic–immune heterogeneity of sepsis and highlight GYG1 as a promising therapeutic target.

## Materials and methods

### Bulk RNA-seq data acquisition and processing

Gene expression data of sepsis patients were retrieved from the GEO database (GSE57065) (16) and normalized using the Robust Multi-array Average (RMA) algorithm in R. Differentially expressed genes (DEGs) at 24 h and 48 h post-disease onset were identified relative to baseline (0 h) using the “limma” package (17), with |log2 fold change| > 1 and adjusted p < 0.05 as cutoffs. A list of metabolism-related genes (MRGs) was compiled from MSigDB metabolic gene sets and relevant literature (18), and the intersection between DEGs and MRGs was calculated. Heatmaps of intersected MRGs were generated using the “pheatmap” package with hierarchical clustering (19).

### Consensus clustering and immune infiltration analysis

Patients were classified into metabolic subgroups based on the expression of intersected MRGs using consensus clustering (ConsensusClusterPlus) (20). Immune cell composition was estimated via the CIBERSORT algorithm (21), and differences between subgroups were assessed by Wilcoxon rank-sum test. Gene Set Enrichment Analysis (GSEA) and Gene Ontology (GO) enrichment were performed to explore functional differences between clusters (22, 23). Volcano plots and functional enrichment bubble charts were created in R.

### Machine learning and construction of the metabolic risk score

Least Absolute Shrinkage and Selection Operator (LASSO) regression was applied to DEGs between metabolic clusters to identify hub genes using the “glmnet” package (24). The metabolic risk score was calculated as a weighted sum of hub gene expression, with coefficients derived from LASSO regression. The predictive performance of each gene was evaluated by receiver operating characteristic (ROC) curve analysis in the external dataset GSE95233 (25).

### Single-cell RNA-seq data processing and annotation

Single-cell transcriptomic data of peripheral blood mononuclear cells from sepsis patients and healthy controls were obtained from published datasets GSE167363 (26). Data were processed using Seurat in R (27), including quality filtering, normalization, scaling, and principal component analysis. Cells were clustered via graph-based methods and visualized using Uniform Manifold Approximation and Projection (UMAP) (28). Cell types were annotated with canonical marker genes, and platelet/erythroid lineages were excluded from downstream analyses. The distribution of immune subsets was compared between high- and low-risk groups. Cell clusters were annotated based on the expression of canonical marker genes. Clusters exhibiting dominant expression of platelet markers (PPBP, PF4) or erythroid markers (HBB) were excluded from subsequent analysis to focus on nucleated immune cell populations. Hub gene expression patterns across immune cell subsets were assessed using violin plots.

### Cell–cell communication analysis

Cell–cell interaction networks were inferred using the CellChat package (29). Ligand–receptor interaction frequencies and strengths were compared between high- and low-risk groups. Differential communication patterns were visualized via heatmaps and network diagrams, with a focus on monocyte–dendritic cell interactions.

### *In vivo* validation and LNP–siRNA delivery

Eight-week-old female C57BL/6 mice were used for an LPS-induced sepsis model. Mice were randomly assigned to control, LNP-control siRNA, and LNP–siGyg1 groups (n = 10 per group). Lipid nanoparticles (LNPs) containing siRNA targeting Gyg1 were formulated using DLin-MC3-DMA as the cationic lipid component (30). LNP–siGyg1 was administered intravenously 24 hours prior to LPS injection (10 mg/kg, intraperitoneal) and repeated every 24 h. SiRNA targeting Gyg1 was acquired from Thermo Fisher. qPCR was performed on peripheral blood samples to measure hub gene expression. All qPCR analyses in this study were normalized to β-actin, which served as the internal reference gene. The relative mRNA expression levels were calculated using the 2^–ΔΔCt method. Total leukocytes (RBC-lysed peripheral blood cells) were collected from mice treated with either control LNPs or LNP–siGyg1. Flow cytometric sorting was subsequently performed to purify monocytes (CD11b^+^Ly6G^−^) and neutrophils (Ly6G^+^) from the same samples. Survival was monitored for every 40 hours post-LPS injection, and differences were analyzed by Kaplan–Meier curves. All mice were obtained from The Jackson Laboratory and housed under specific pathogen-free conditions at the Zunyi Medical University animal facility. All animal procedures were approved by the Zunyi Medical University ethics committee with the approved number (2024) 1-068.

### Flow cytometry, glycogen quantification, serum cytokine ELISA and cell migration assay

Peripheral blood cells were collected into heparinized tubes and subjected to red blood cell lysis (ACK buffer, Thermo Fisher). Total leukocytes were stained with fluorescent antibodies against CD45, CD11b, Ly6G, CD11c, MHC-II, CD4, CD8, NK1.1, and B220 (BioLegend). The markers are as follows: Neutrophils (CD11b^+^ Ly6G^+^), Monocytes (CD11b^+^ Ly6G^−^), Dendritic cells (CD11c^+^), T cells (CD4^+^ or CD8^+^), B/NK cells (B220^+^ or NK1.1^+^). Flow cytometry and sorting were performed on BD FACSAria III. Data were analyzed with FlowJo v10.

As for glycogen quantification, Sorted neutrophils and monocytes were lysed, and glycogen content was determined using a Glycogen Assay Kit (Sigma-Aldrich, MAK016) following the manufacturer’s instructions. Absorbance was measured at 570 nm, and results were normalized to cell number and expressed as µg/µl glycogen. Serum levels of IL-6, TNF-α, and IL-1β were quantified 6h after LPS treatment using mouse ELISA kits (R&D Systems) according to the manufacturer’s protocol. Cytokine concentrations were determined by standard curves and expressed in pg/mL.

To assess the migratory capacity of immune cells after Gyg1 knockdown, neutrophils and monocytes were isolated from peripheral blood of LNP–siGyg1– or LNP–ctrl–treated mice using FACS sorting. Migration assays were performed using 24-well Transwell chambers with 5-µm pore polycarbonate membranes (Corning, 3421). Cells (1 million per insert) were suspended in serum-free RPMI-1640 medium and placed in the upper chamber, while the lower chamber contained medium supplemented with CCL2 (100 ng/mL) for monocytes or CXCL1 (100 ng/mL) for neutrophils (PeproTech). After incubation for 30 min or 3 hours at 37 °C, cells that migrated to the lower chamber were collected and counted using a hemocytometer or flow cytometry. Migration efficiency was expressed as the percentage of input cells migrated. All experiments were performed in triplicate.

### Statistical analysis

Statistical analyses were performed using R (v4.1.2) and Python (v3.8). Data are expressed as mean ± standard deviation (SD). Two-group comparisons were conducted with Wilcoxon rank-sum tests, while multi-group comparisons used one-way ANOVA followed by Tukey’s *post hoc* test. Survival differences were assessed by log-rank test. A p-value < 0.05 was considered statistically significant.

## Results

### Identification and characterization of metabolism-related genes in sepsis

**Figure 1:**
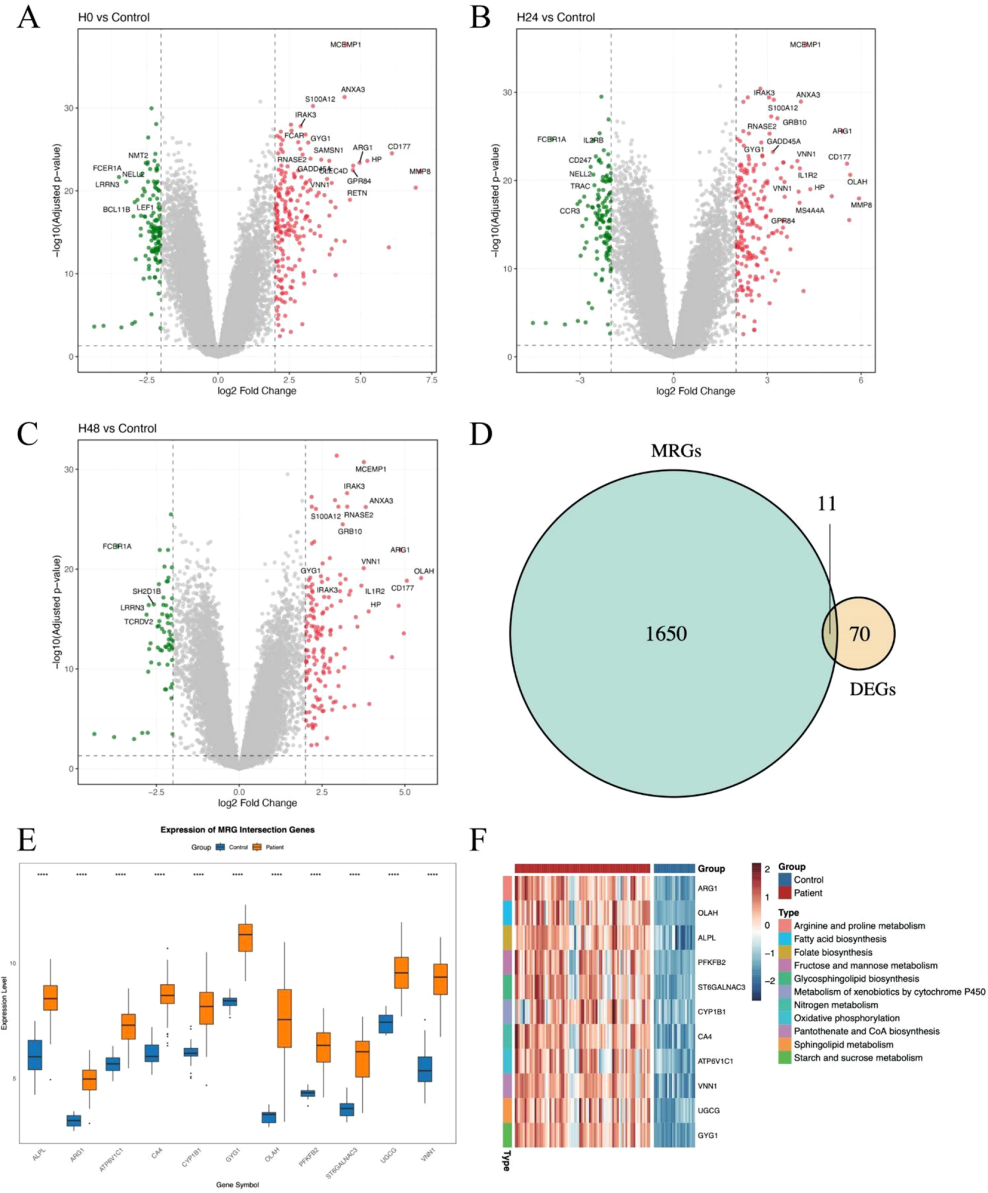
Identification and characterization of metabolism-related genes in sepsis. **(A–C)** Volcano plots showing differentially expressed genes (DEGs) between sepsis samples at 0h, 24 h and 48 h. Significantly upregulated and downregulated genes are highlighted. **(D)** Venn diagram showing the intersection between DEGs and metabolism-related genes (MRGs) curated from public databases and literature. **(E)** Boxplot showing the average expression of the 11 DEGs between patients and healthy controls. **(F)** Heatmaps showing the expression of intersected MRGs across sepsis samples at 0 h, 24 h, and 48 h. Each column represents a patient sample, and each row represents a gene. Hierarchical clustering was applied to both genes and samples. The symbol “****” represents a p-value < 0.0001, indicating a highly statistically significant difference.

To systematically explore the role of metabolism-related genes (MRGs) in sepsis, we first analyzed transcriptional changes at three time points—0 h, 24 h, and 48 h—using the whole blood transcriptomic dataset GSE57065. Differential expression analysis was conducted by comparing patient samples to healthy samples (**Figure 1A–C**). A total of 70 differentially expressed genes (DEGs) were identified across all time points, indicating a dynamic transcriptomic response during the early progression of sepsis. To specifically evaluate the contribution of metabolic pathways, we intersected these DEGs with a curated list of metabolism-related genes, yielding a panel of metabolic DEGs with potential relevance to sepsis pathophysiology (**Figure 1D**). Notably, many of these genes are involved in key processes such as mitochondrial function, glycolysis, and amino acid metabolism. We next visualized the expression of these metabolic DEGs across all patient samples (**Figure 1E**). Heatmap clustering analysis revealed that several genes exhibited consistent temporal trends, suggesting potential regulatory programs associated with metabolic reprogramming in sepsis (**Figure 1F**). These genes may serve as candidate biomarkers or therapeutic targets, warranting further validation.

### Metabolism-related gene expression stratifies sepsis patients into distinct immunological subgroups

**Figure 2:**
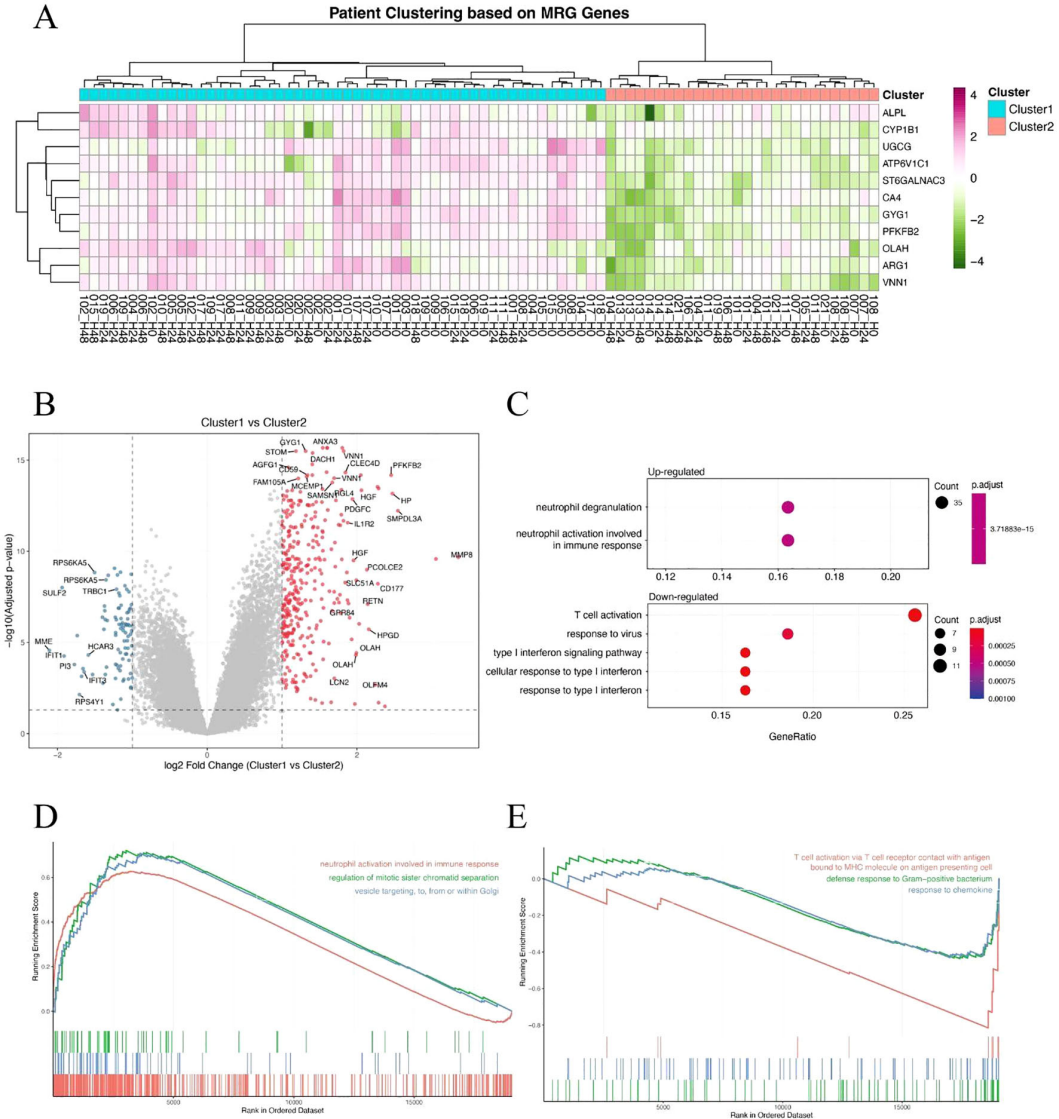
Metabolism-related gene expression stratifies sepsis patients into distinct immunological subgroups. **(A)** Hierarchical clustering of sepsis patients based on metabolism-related gene (MRG) expression, revealing two clusters (Cluster 1 and Cluster 2). **(B)** Volcano plot showing differentially expressed genes (DEGs) between Cluster 1 and Cluster 2. Red and blue dots represent significantly upregulated and downregulated genes, respectively. **(C)** GO biological process enrichment analysis of DEGs, showing neutrophil-associated immune responses enriched in Cluster 1. **(D, E)** Gene Set Enrichment Analysis (GSEA) of hallmark or GO terms between the two clusters. Cluster 1 shows enrichment in neutrophil activation and antibacterial responses **(D)**, while Cluster 2 is associated with T cell-mediated immune pathways **(E)**.

To determine whether metabolism-related genes (MRGs) could distinguish biologically distinct subgroups of sepsis patients, we performed unsupervised hierarchical clustering based on the expression of previously identified MRGs. This approach stratified patients into two major clusters, designated Cluster 1 and Cluster 2 (**Figure 2A**). The clustering revealed substantial transcriptional heterogeneity, suggesting differential metabolic states among patients. We then identified differentially expressed genes (DEGs) between the two clusters to investigate their molecular differences. A large number of DEGs were observed, with both significantly upregulated and downregulated genes distinguishing the two groups (**Figure 2B**). Subsequent Gene Ontology (GO) enrichment analysis of the DEGs revealed that Cluster 1 was characterized by enrichment in a neutrophil-dominant immune response state (**Figure 2C**). Furthermore, Gene Set Enrichment Analysis (GSEA) reinforced these findings: Cluster 1 showed significant enrichment in neutrophil responses and defense against pathogens (**Figure 2D**), whereas Cluster 2 was more enriched in T cell responses (**Figure 2E**). Together, these findings suggest that metabolism-based clustering captures biologically meaningful immunological differences and may help explain the variable clinical outcomes observed in sepsis.

### Identification of hub genes and construction of a metabolic risk score model in sepsis

**Figure 3:**
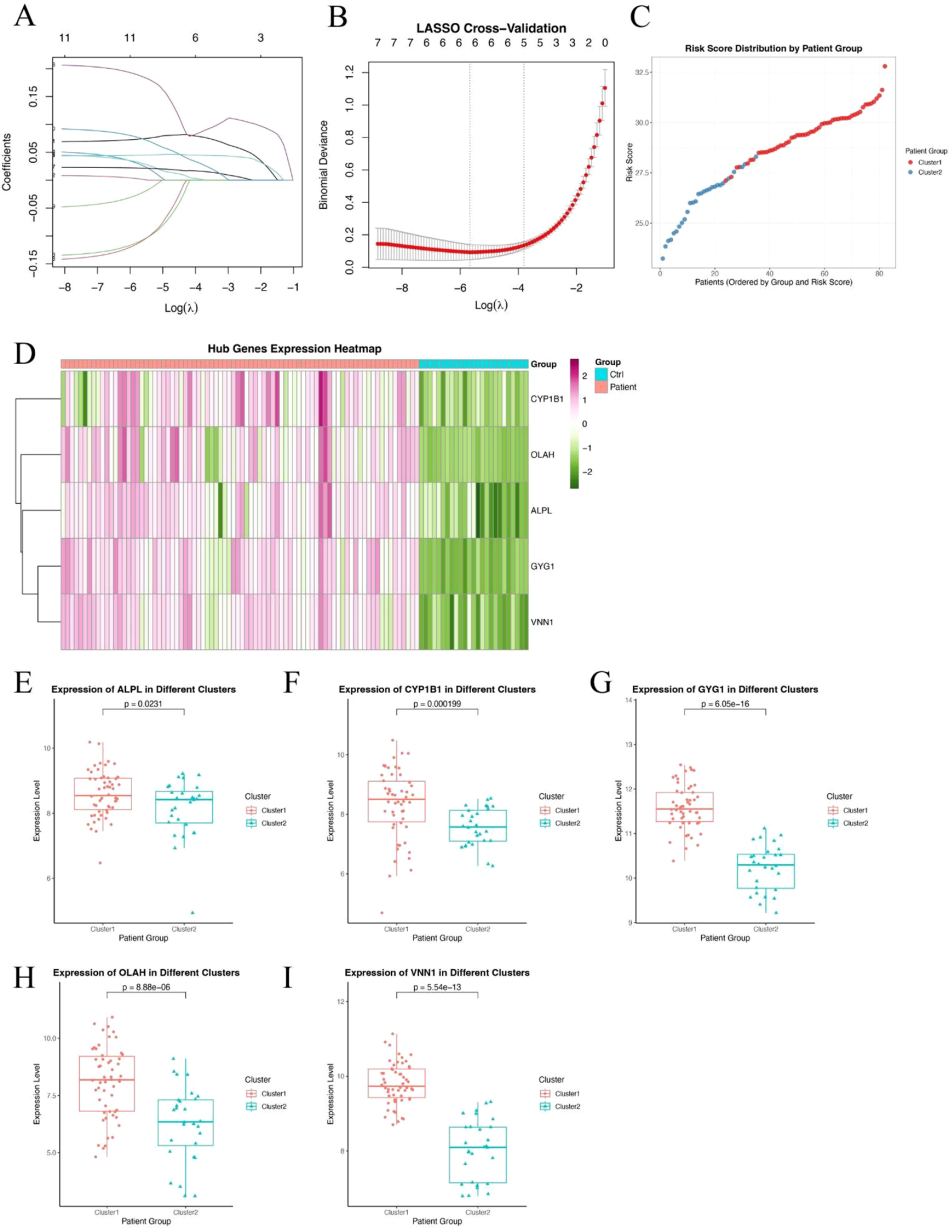
Identification of hub genes and construction of a metabolic risk score model. **(A, B)** LASSO regression and cross-validation plots based on 11 DEGs between Cluster 1 and Cluster 2. Five hub genes were selected based on the optimal lambda value. **(C)** Distribution of the calculated risk score across patients from Cluster 1 and Cluster 2, showing higher scores in neutrophil-dominant Cluster 1. **(D)** Heatmap showing expression patterns of the five hub genes (ALPL, CYP1B1, GYG1, OLAH, VNN1) across all sepsis samples. **(E–I)** Box plots showing significantly higher expression levels of ALPL **(E)**, CYP1B1 **(F)**, GYG1 **(G)**, OLAH **(H)**, and VNN1 **(I)** in Cluster 1 compared to Cluster 2. P-values are indicated above each comparison.

To further refine clinically relevant metabolic signatures in sepsis, we applied a machine learning approach to the 11 differentially expressed genes (DEGs) identified between Cluster 1 and Cluster 2. Least absolute shrinkage and selection operator (LASSO) regression was performed to reduce feature redundancy and select the most informative genes (**Figures 3A, B**). Five hub genes—ALPL, CYP1B1, GYG1, OLAH, and VNN1 were identified as optimal predictors based on the minimum binomial deviance criteria. We then constructed a metabolic risk score model based on the expression levels of these five genes. The risk score formula was defined as: Risk score = 0.231 × ALPL + 0.218 × CYP1B1 + 1.930 × GYG1 + 0.0829 × OLAH + 0.277 × VNN1. This score effectively stratified patients from Cluster 1 and Cluster 2, with significantly higher scores observed in patients from the neutrophil-dominant Cluster 1 (**Figure 3C**), indicating that the hub gene signature captures cluster-specific immunometabolic phenotypes. To further examine the expression patterns of the hub genes, we visualized their expression in all samples (**Figure 3D**). Notably, all five hub genes were significantly upregulated in Cluster 1 compared to Cluster 2 (**Figures 3E–I**), suggesting that elevated expression of these genes is associated with the hyperinflammatory metabolic state seen in neutrophil-dominant sepsis subtypes.

### Hub genes demonstrate robust and accurate diagnostic performance in an independent validation cohort

**Figure 4:**
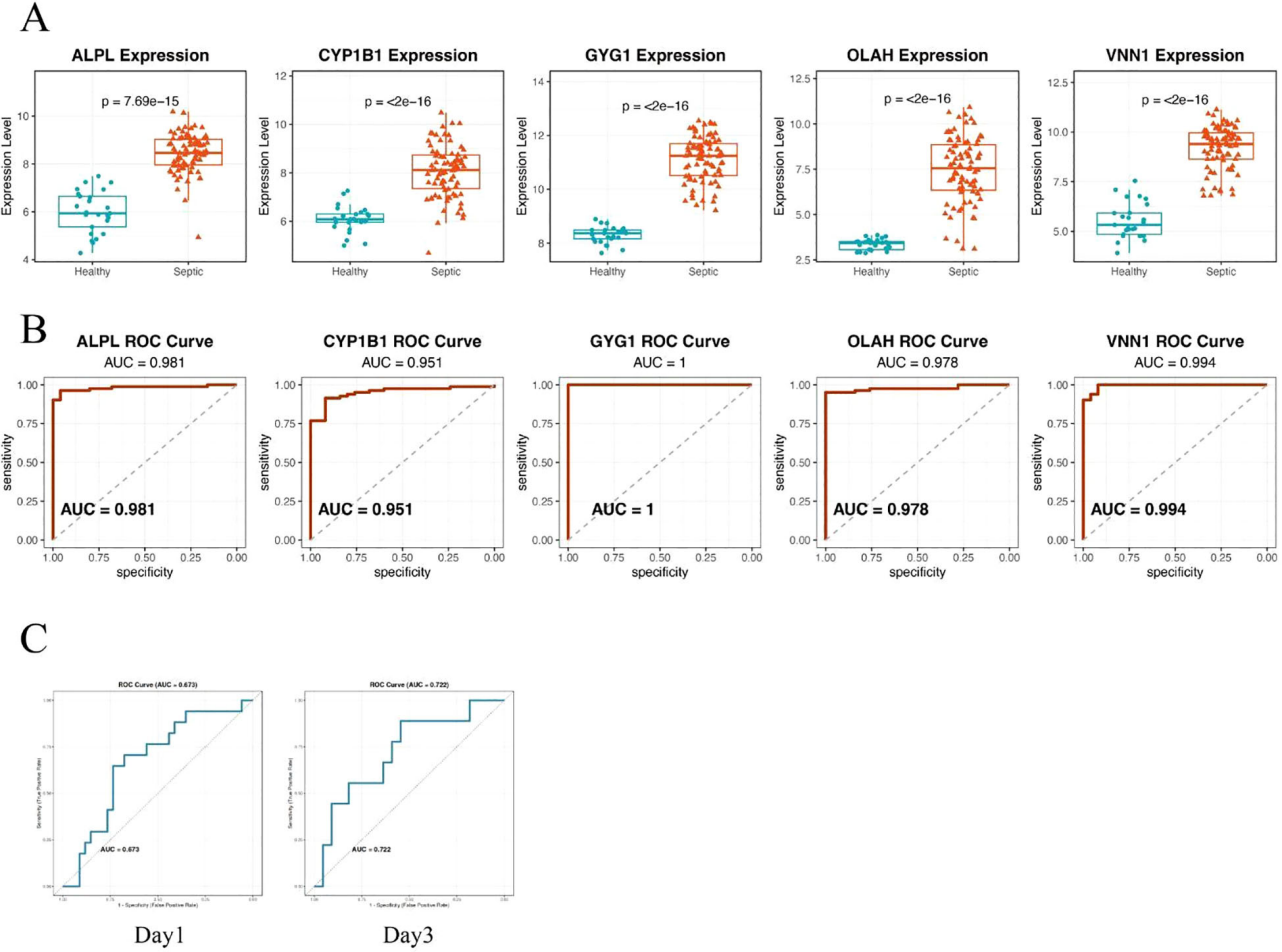
Hub genes demonstrate robust and accurate diagnostic performance in an independent validation cohort. **(A)** Box plots showing significantly higher expression levels of the five hub genes—ALPL, CYP1B1, GYG1, OLAH, and VNN1—in septic patients compared to healthy controls. **(B)** ROC curve analysis demonstrating the diagnostic performance of each gene in distinguishing sepsis from healthy controls. All five genes exhibited high predictive accuracy, with AUC values exceeding 0.95. **(C)** ROC curve of the metabolic risk score model in predicting the survival of sepsis patients based on the blood samples of day 1(left) or day 3(right).

To validate the diagnostic potential of the five identified hub genes, we applied the model to an independent external dataset (GSE95233, **Supplementary Table 1**), which includes peripheral blood samples from septic patients and healthy controls. All five hub genes—ALPL, CYP1B1, GYG1, OLAH, and VNN1—were significantly upregulated in septic patients compared to healthy individuals (**Figure 4A**), consistent with findings from the discovery cohort. We further evaluated the predictive performance of each gene using receiver operating characteristic (ROC) curve analysis. Strikingly, all five genes demonstrated excellent discriminative power, with area under the curve (AUC) values of 0.981 for ALPL, 0.951 for CYP1B1, 1.000 for GYG1, 0.978 for OLAH, and 0.994 for VNN1, respectively (**Figure 4B**). The consistently high AUC values suggest that this metabolic gene signature may serve as a robust biomarker panel for sepsis detection. It is worth noting that the perfect classification performance of GYG1 (AUC = 1.0) in the validation cohort may be partly attributed to the limited sample size and needs to be interpreted with caution. Nonetheless, these findings support the strong diagnostic relevance of the identified hub genes. Our model also achieved an AUC of 0.673 (Day 1) and 0.722 (Day 3) in predicting the survival of the sepsis patients in the external validation cohort (new **Figure 4C**). This performance is comparable to the reported efficacy of gold-standard clinical scores like SOFA and APACHE II from large-scale studies, which typically show AUCs in the range of 0.65-0.75 for survival prediction (31, 32).

### High metabolic risk score in sepsis is associated with neutrophil and monocyte-dominant and lymphocyte-suppressed immune infiltration

**Figure 5:**
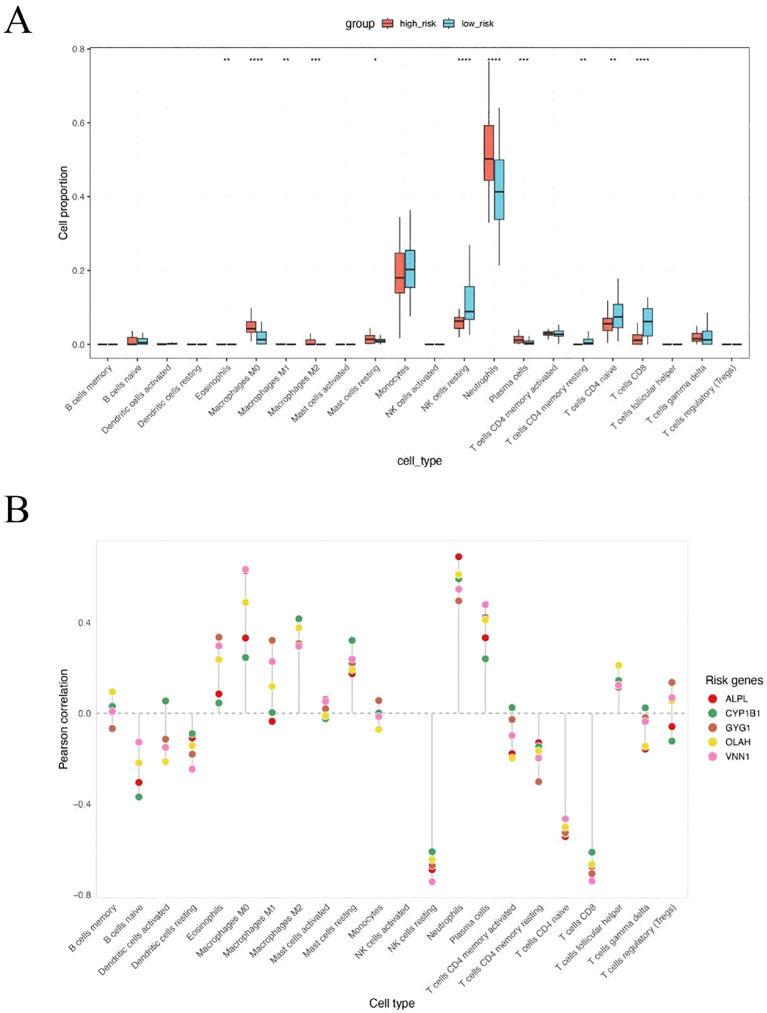
High metabolic risk score in sepsis is associated with neutrophil and monocyte-dominant and lymphocyte-suppressed immune infiltration. **(A)** CIBERSORT-based estimation of immune cell infiltration in high-risk versus low-risk patients. Neutrophils were significantly increased in high-risk patients, while NK cells, CD4+ T cells, and CD8+ T cells were significantly decreased. **(B)** Pearson correlation analysis between expression levels of the five hub genes and immune cell fractions. All five genes were positively correlated with neutrophils, monocytes, and macrophages, and negatively correlated with T cells and NK cells. The symbols represent the following levels of statistical significance: *: p < 0.05; **: p < 0.01; ***: p < 0.001; ****: p < 0.0001.

To explore the immunological differences between metabolic subgroups of sepsis, we investigated the immune cell infiltration landscape of high- and low-risk patients defined by our five-gene risk score model. Using the CIBERSORT deconvolution algorithm, we estimated the relative proportions of 22 immune cell types across all patients. Significant differences in immune infiltration were observed between the two groups (**Figure 5A**). High-risk patients exhibited markedly elevated levels of neutrophils and monocytes, alongside reduced proportions of natural killer (NK) cells and multiple T cell subpopulations, including CD4+ and CD8+ T cells. These findings suggest that high metabolic risk in sepsis is associated with a neutrophil-dominant, lymphocyte-suppressed immune landscape. To further elucidate the immunological relevance of each hub gene, we performed Pearson correlation analysis between gene expression levels and estimated immune cell fractions (**Figure 5B**). All five risk genes—ALPL, CYP1B1, GYG1, OLAH, and VNN1—showed strong positive correlations with neutrophils, monocytes, and macrophages, and consistent negative correlations with T cells and NK cells. These results suggest that the severity of sepsis may be linked to an imbalance between innate and adaptive immune responses, with high-risk patients exhibiting a shift toward an innate immunity-dominated profile.

### Single-cell analysis of immune cell composition in septic patients

**Figure 6:**
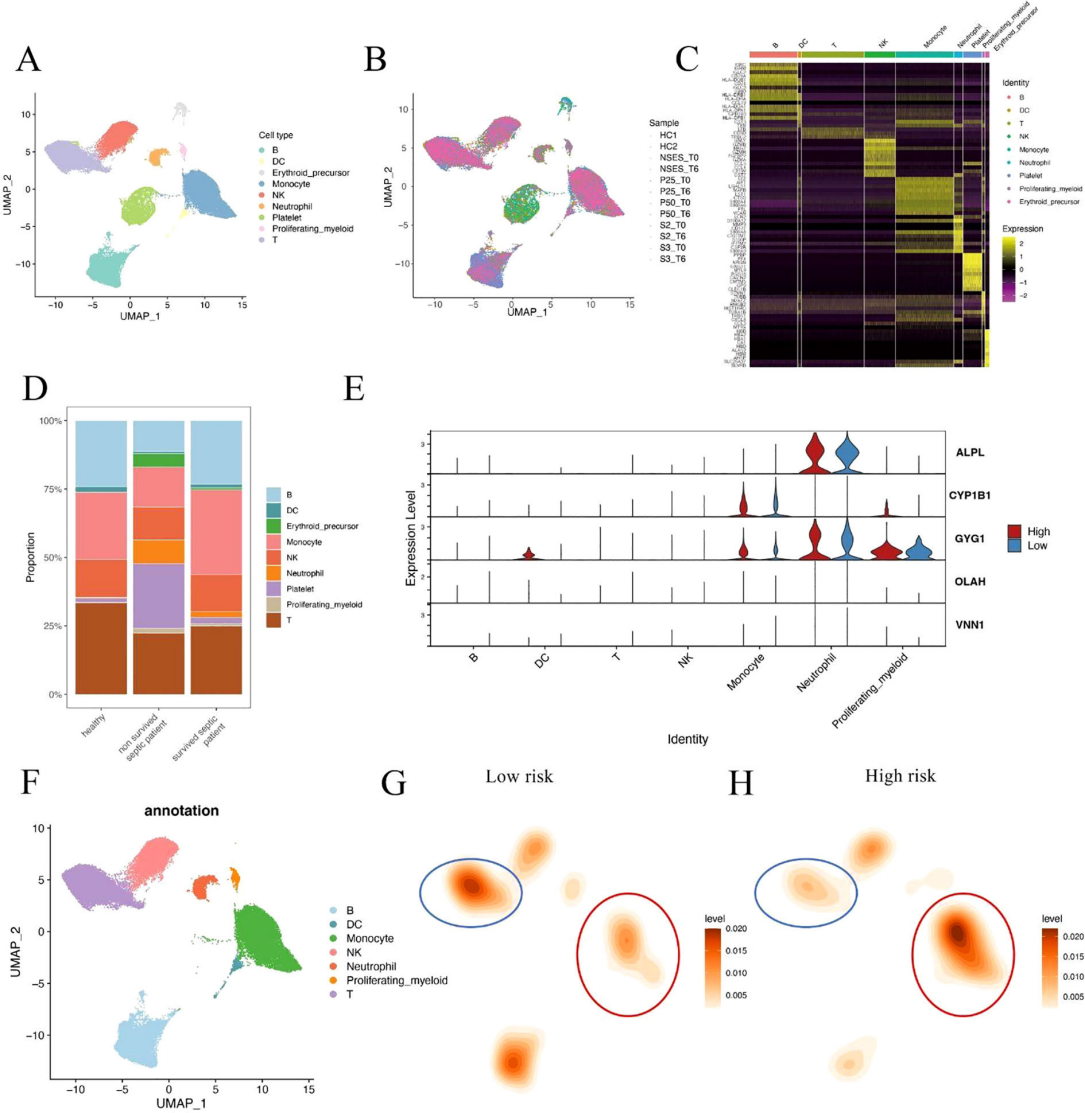
Single-cell analysis of immune cell composition in septic patients. **(A, B)** UMAP visualization of integrated single-cell RNA-seq data from septic patients and healthy controls. Cells were clustered and annotated based on canonical immune markers. Platelet and erythroid cells were excluded from downstream analysis. **(C)** Heatmap of representative marker gene expression used for immune cell type annotation. **(D)** Cell composition comparison across clinical groups shows a significantly increased neutrophil fraction in non-surviving sepsis patients relative to survivors and healthy controls. **(E)** Violin plots showing expression of hub genes (ALPL, CYP1B1, GYG1, OLAH, VNN1) across immune cell subsets in high- and low-risk patients. Three genes were enriched in innate immune cells, while two showed minimal expression in immune lineages. **(F)** UMAP plot showing immune cell type annotations used in downstream comparison. **(G, H)** Density maps illustrating differences in immune cell distributions between low-risk **(G)** and high-risk **(H)** patients. Monocytes were expanded in high-risk patients, while T cell density was reduced.

To further validate the association between metabolic risk and immune cell remodeling in sepsis, we performed integrative single-cell transcriptomic analysis using published peripheral blood mononuclear cell (PBMC) datasets. After standard quality control, normalization, and dimensionality reduction, immune cells were clustered and visualized using UMAP (**Figures 6A, B**). Cell identities were annotated based on canonical marker genes, and platelet/erythroid lineage cells were excluded to focus on immunologically relevant populations (**Figure 6C**). Comparative analysis of cell composition across clinical groups revealed that non-surviving septic patients exhibited a markedly higher proportion of neutrophils compared to both healthy controls and survivors (**Figure 6D**). This observation is consistent with our earlier findings based on CIBERSORT deconvolution of bulk transcriptomic data, reinforcing the association between neutrophil and innate immune dominance and poor clinical outcome in sepsis. To further investigate the cellular origin of the risk score components, we analyzed the expression patterns of the five hub genes across different immune subsets (**Figure 6E**). Three of the five genes—ALPL, CYP1B1, and GYG1—were predominantly expressed in monocytes, neutrophils, and proliferating myeloid cells, consistent with their potential roles in innate immune activation. In contrast, OLAH and VNN1 showed low expression across all major immune cell types, suggesting that these genes may be expressed in non-immune blood components (e.g., endothelial cells or platelets) and contribute to sepsis progression through indirect mechanisms.

To better characterize the immune context of metabolic risk in sepsis, we compared the single-cell distribution patterns between high- and low-risk patients. Immune cells were first annotated based on canonical marker expression (**Figure 6F**). Density estimation maps revealed that high-risk patients showed marked expansion of monocytes, along with a notable reduction in T cells compared to the low-risk group (**Figures 6G, H**). These findings reinforce the hypothesis that severe sepsis is associated with a shift toward innate immunity dominance, coupled with adaptive immune suppression. These results suggest that innate immune hyperactivation, particularly neutrophil and monocyte expansion, may play a critical role in driving sepsis severity and mortality.

### Single-cell communication analysis reveals intensified monocyte–DC interactions in high-risk sepsis patients

**Figure 7:**
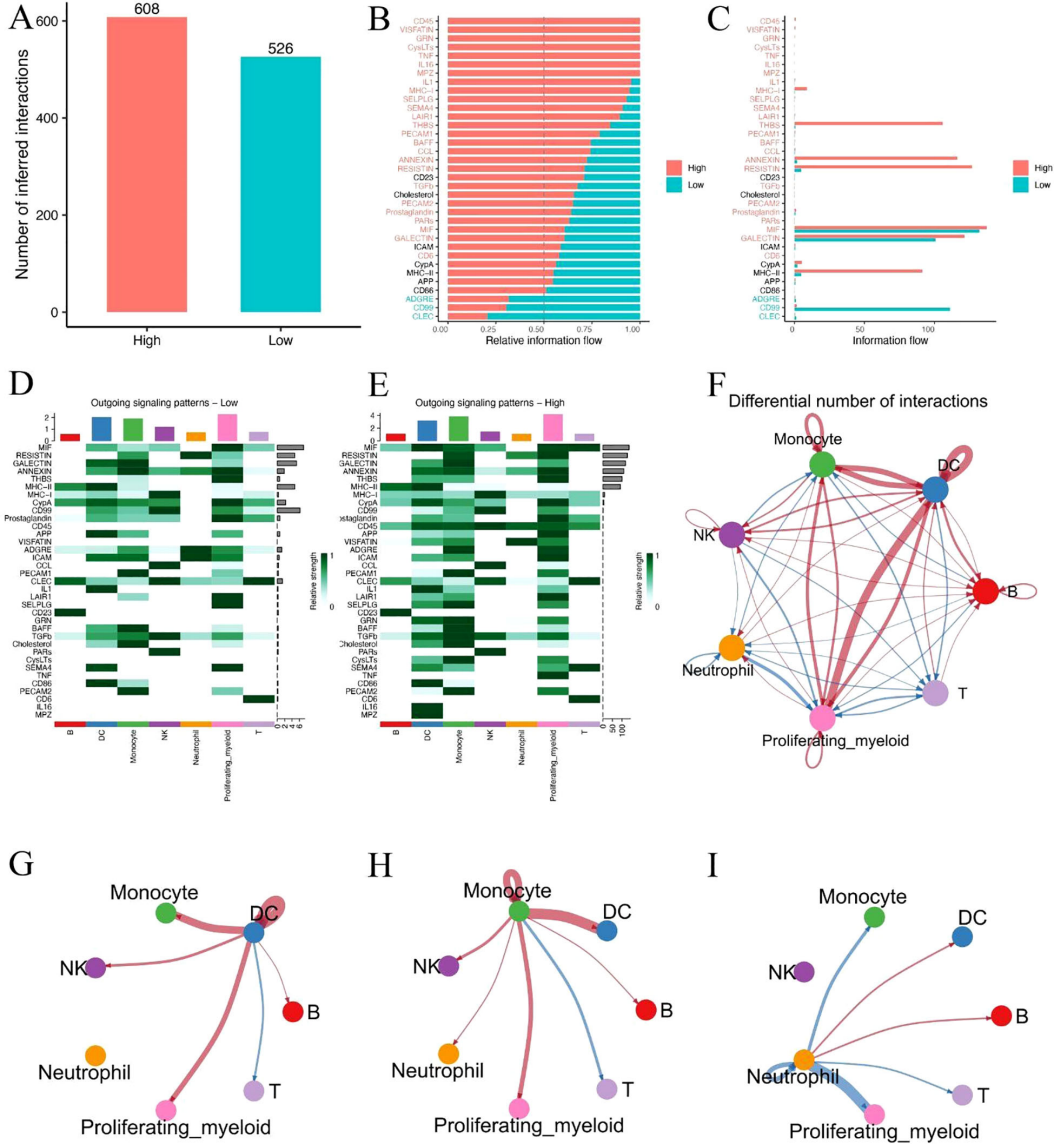
Single-cell communication analysis reveals intensified monocyte–DC interactions in high-risk sepsis patients. **(A)** Total number of predicted ligand–receptor interactions in high- and low-risk groups. **(B, C)** Relative and absolute information flow across signaling pathways in both groups. **(D, E)** Heatmaps showing outgoing signaling strength from each cell type in low-risk **(D)** and high-risk **(E)** patients. **(F)** Overall cell–cell communication network based on differential interaction strength. **(G–I)** Focused visualization of directed intercellular signaling networks, highlighting intensified monocyte–DC interactions in the high-risk group.

To investigate potential alterations in intercellular communication associated with metabolic risk, we performed ligand–receptor interaction inference based on single-cell expression profiles in high- and low-risk sepsis patients. A total of 608 inferred interactions were detected in the high-risk group, compared to **526** in the low-risk group (**Figure 7A**), indicating a globally enhanced signaling environment in high-risk patients. Relative information flow analysis revealed that high-risk patients exhibited greater activity across a range of signaling pathways (**Figures 7B, C**), particularly those associated with inflammation and antigen presentation (e.g., CD40, MIF, SELPLG). In contrast, several homeostatic or regulatory pathways (e.g., CCL, CD46) were more active in the low-risk group. Heatmap visualization of outgoing signaling patterns further demonstrated that high-risk patients had enhanced pathway activity originating from monocytes and dendritic cells (DCs) (**Figures 7D, E**). Network topology analysis revealed a dense interaction hub centered around these two cell types in high-risk patients, with significantly elevated communication frequency and intensity compared to low-risk individuals (**Figure 7F**). Notably, focused cell–cell interaction mapping confirmed that monocyte–DC signaling was markedly stronger in the high-risk group (**Figures 7G–I**). These findings suggest that increased monocyte-DC crosstalk may contribute to the hyperinflammatory immune environment and disease progression in metabolically high-risk sepsis patients.

### GYG1 is highly expressed in innate immune cells and associated with pro-inflammatory transcriptional programs in high-risk patients

**Figure 8:**
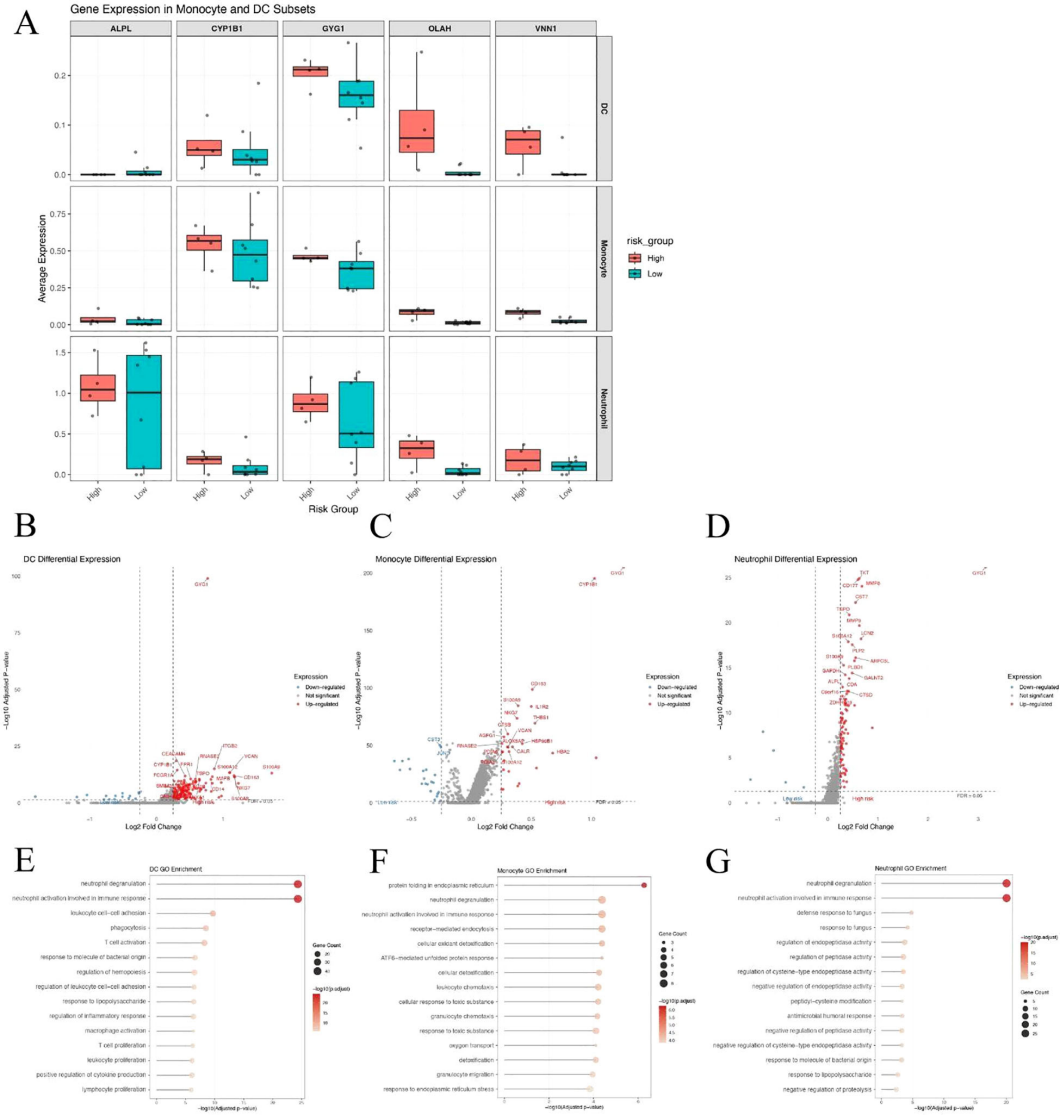
GYG1 is highly expressed in innate immune cells and associated with pro-inflammatory transcriptional programs in high-risk patients. **(A)** Box plots showing expression of five hub genes in dendritic cells (DCs), monocytes, and neutrophils from high- and low-risk sepsis patients. GYG1 is consistently expressed and upregulated across all three cell types. **(B–D)** Volcano plots of differentially expressed genes in DCs **(B)**, monocytes **(C)**, and neutrophils **(D)** between high- and low-risk patients. **(E–G)** GO enrichment analysis of genes upregulated in the high-risk group in DCs **(E)**, monocytes **(F)**, and neutrophils **(G)**, showing shared enrichment in neutrophil degranulation and innate immune activation pathways.

Given the substantial differences in dendritic cells (DCs), monocytes, and neutrophils between high- and low-risk patients, we examined the expression of the five hub genes across these three cell types. Among them, GYG1 was consistently expressed in all three cell populations and showed significantly higher expression in the high-risk group (**Figure 8A**), suggesting that it may play a key role in orchestrating innate immune activation in metabolically high-risk patients. We next performed differential gene expression (DEG) analysis between high- and low-risk patients within each of the three cell types. The volcano plots revealed a large number of upregulated genes in high-risk DCs (**Figure 8B**), monocytes (**Figure 8C**), and neutrophils (**Figure 8D**), many of which are associated with innate immune functions. Gene Ontology enrichment analyses of the upregulated DEGs in each cell type showed consistent results: neutrophil degranulation and activation-related pathways were significantly enriched in all three cell populations (**Figures 8E–G**). These findings suggest that GYG1 may be involved in driving pro-inflammatory transcriptional programs in key innate immune subsets, potentially contributing to disease severity in high-risk sepsis patients.

### LNP-mediated Gyg1 silencing improves survival in LPS-induced sepsis mouse model

**Figure 9:**
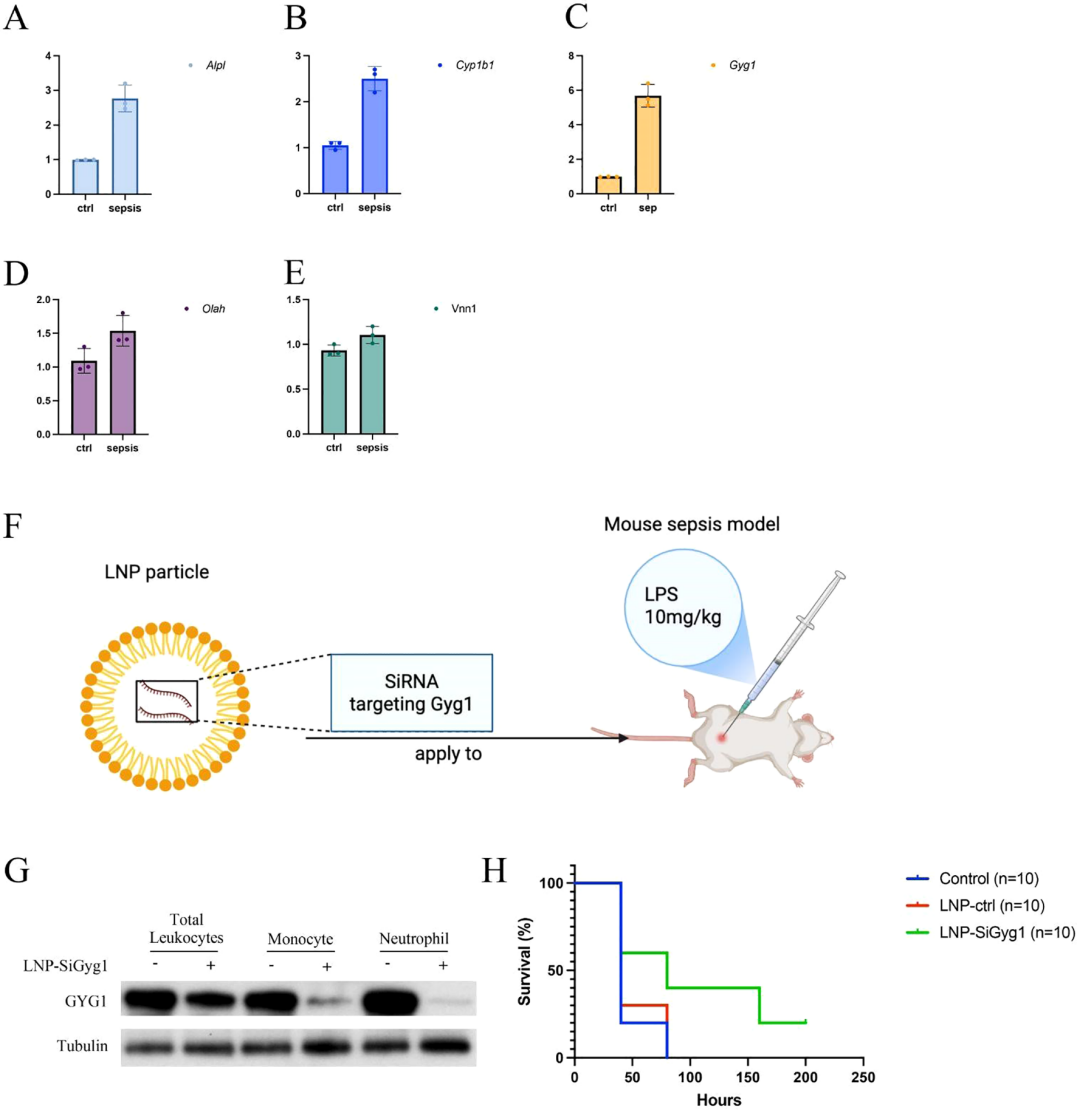
LNP-mediated Gyg1 silencing improves survival in an LPS-induced sepsis mouse model. **(A–E)** qPCR analysis of hub gene expression in peripheral blood from control and LPS-induced sepsis mice. Gyg1 exhibited the most pronounced upregulation among the five hub genes. **(F)** Schematic of LNP formulation and dosing strategy for Gyg1 siRNA delivery in the LPS sepsis model. LNPs were administered intravenously 24 h before and every 24 h after LPS injection (10 mg/kg). **(G, H)** Kaplan–Meier survival curves showing improved survival in the LNP-SiGyg1 group compared to control and LNP-control siRNA groups.

To validate the *in vivo* relevance of the hub genes identified in our computational analyses, we first measured their expression in peripheral blood from an LPS-induced sepsis mouse model using qPCR. Among the five hub genes (Alpl, Cyp1b1, Gyg1, Olah, Vnn1), all except Olah and Vnn1 were significantly upregulated in septic mice compared to controls, with Gyg1 showing the most pronounced increase (**Figures 9A–E**). Given the strong predictive performance of Gyg1 and its association with pro-inflammatory innate immune programs, we next investigated whether targeting Gyg1 could ameliorate sepsis outcomes. We formulated lipid nanoparticles (LNPs) encapsulating siRNA specific to Gyg1 (LNP-SiGyg1) and administered them intravenously 24 hours prior to LPS injection (10 mg/kg) and at 24-hour intervals thereafter (**Figure 9F**). To confirm the cellular specificity and efficiency of LNP–siGyg1 delivery, we evaluated GYG1 protein expression in distinct peripheral immune subsets. Western blot analysis demonstrated a marked reduction of GYG1 protein in both monocytes and neutrophils following LNP–siGyg1 treatment, whereas total leukocytes also exhibited an overall decrease, confirming effective knockdown of Gyg1 in circulating myeloid cells (**Figure 9G**). Kaplan–Meier survival analysis revealed that Gyg1 silencing markedly improved survival compared to both untreated and LNP-control siRNA-treated groups (**Figure 9H**), suggesting that metabolic targeting of Gyg1 may offer therapeutic benefit in sepsis.

### GYG1 knockdown reduces glycogen content and inflammatory activation in myeloid cells *in vivo*

**Figure 10:**
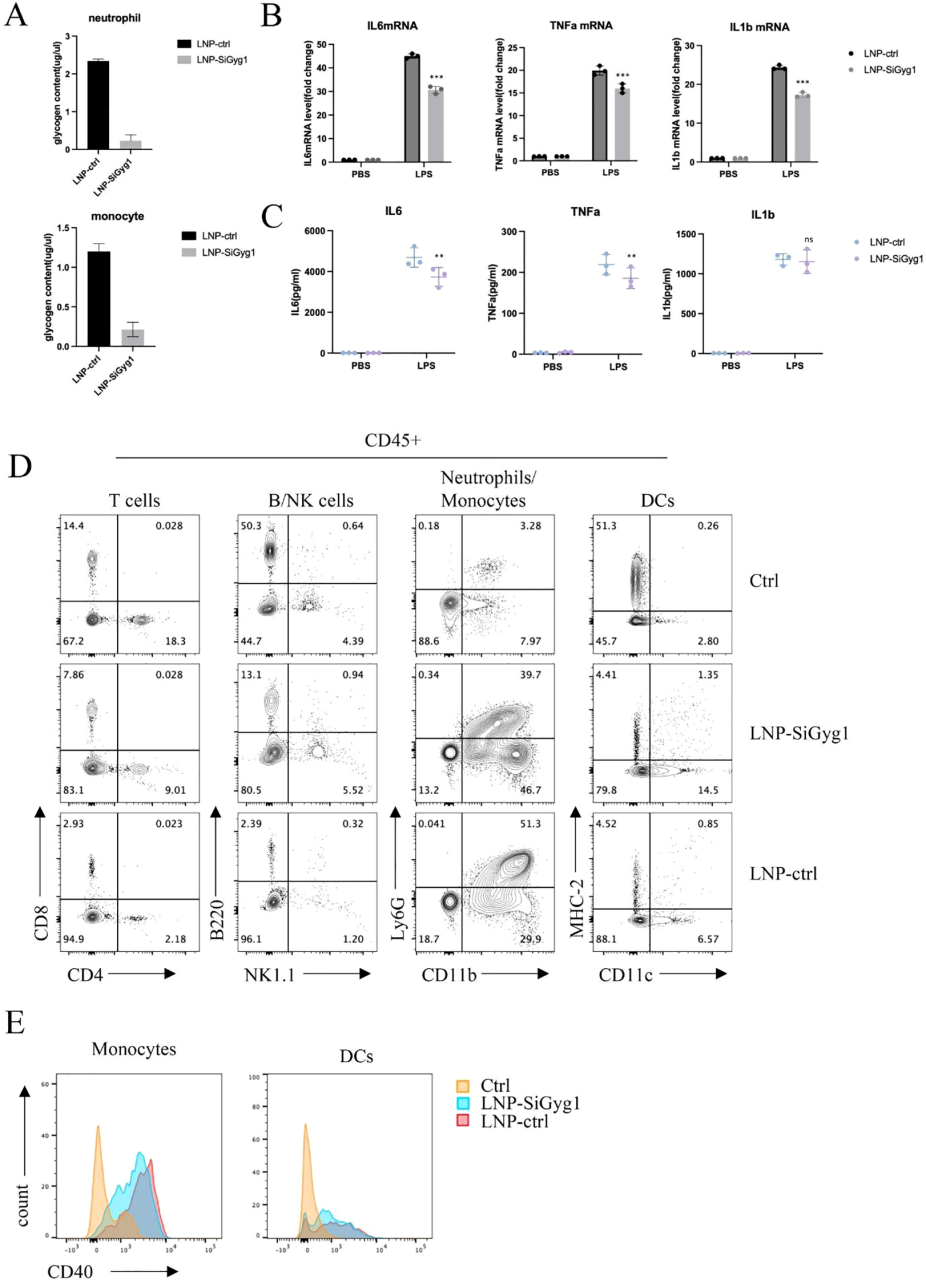
GYG1 knockdown reduces glycogen metabolism and inflammatory activation in myeloid cells *in vivo*. **(A)** Quantification of glycogen content in sorted neutrophils and monocytes from mice treated with LNP–siGyg1 or control LNPs. GYG1 knockdown significantly decreased intracellular glycogen levels in both cell types. **(B)** Relative mRNA expression of IL6, TNFA, and IL1B in peripheral monocytes and neutrophils from LPS-challenged mice (6h post LPS inject) with or without GYG1 silencing. Data were normalized to β-actin. **(C)** Serum concentrations of IL-6, TNF-α, and IL-1β measured by ELISA (6h post LPS inject). GYG1 knockdown markedly reduced IL-6 and TNF-α levels, with minor effects on IL-1β. **(D)** Flow cytometry profiling of major immune subsets (T cells, B/NK cells, neutrophils/monocytes, and dendritic cells) in septic mice treated with control or GYG1-targeting LNPs. GYG1 silencing attenuated LPS-induced neutrophil expansion. **(E)** Flow cytometric analysis of CD40 expression in monocytes and dendritic cells. GYG1 knockdown decreased CD40 expression, suggesting reduced pro-inflammatory activation. Data represent mean ± SD from three independent mice per group. p < 0.05, p < 0.01, p < 0.001 by one-way ANOVA with Tukey’s *post hoc* test.

To further explore the mechanisms of Gyg1 knockdown’s impact on immune metabolism and inflammation, we examined glycogen levels and inflammatory responses in myeloid cells from LPS-induced septic mice treated with LNP–siGyg1. Quantification of glycogen content revealed that Gyg1 silencing significantly reduced intracellular glycogen levels in both neutrophils and monocytes compared with control LNPs (**Figure 10A**), confirming the metabolic efficacy of our knockdown strategy. Next, we evaluated the transcriptional and systemic inflammatory response following Gyg1 inhibition. qPCR analysis showed that Gyg1 knockdown markedly decreased the mRNA expression of IL-6, TNF-α, and IL-1β in peripheral monocytes and neutrophils from LPS-challenged mice (**Figure 10B**). Consistently, ELISA of serum samples demonstrated reduced protein levels of IL-6 and TNF-α, whereas IL-1β levels were not significantly affected (**Figure 10C**).

To further investigate immune cellular changes, we performed flow cytometry profiling of major leukocyte subsets. Gyg1 knockdown strongly attenuated the LPS-induced expansion of neutrophils, while the decreased proportions of T and B/NK cells were partially rescued (**Figure 10D**). Moreover, Gyg1 silencing slightly reduced CD40 expression in both monocytes and dendritic cells (**Figure 10E**), suggesting that diminished glycogen metabolism may suppress pro-inflammatory activation and antigen-presenting potential in myeloid populations. Together, these data demonstrate that Gyg1 depletion alleviates hyperinflammation in sepsis by reducing metabolic fuel availability and dampening myeloid activation.

## Discussion

In this study, we performed a comprehensive analysis of metabolism-related genes (MRGs) in sepsis and developed an immune–metabolic risk score capable of stratifying patients into distinct subgroups with divergent immune landscapes and predicted outcomes. Through machine learning–based feature selection, we identified five hub genes—ALPL, CYP1B1, GYG1, OLAH, and VNN1—that exhibited strong predictive performance, both in the discovery and independent validation cohorts. Single-cell transcriptomic analyses further demonstrated the immune cell–specific distribution of these genes and their correlation with innate and adaptive immune components, providing mechanistic insights into the metabolic–immune heterogeneity observed in sepsis.

Among these genes, GYG1 emerged as a particularly notable candidate. GYG1 encodes glycogenin 1, a core enzyme in glycogen biosynthesis, catalyzing the attachment of glucose residues to a protein primer to initiate glycogen polymerization (33). Beyond its canonical metabolic role, glycogen metabolism has been increasingly recognized as a regulator of immune cell activation, particularly in myeloid cells (34). Our single-cell analysis revealed high expression of GYG1 in monocytes, neutrophils, and proliferating myeloid cells—cell populations that dominate the high-risk, innate immunity–driven sepsis subtype. Functional enrichment analysis of high-risk innate immune cells consistently identified neutrophil degranulation and activation pathways, suggesting that GYG1 may facilitate rapid energy supply to fuel hyperinflammatory responses. The observation that GYG1 knockdown via LNP–siRNA delivery ameliorated disease severity in an LPS-induced sepsis model supports its potential as a metabolic driver of immune dysregulation. Previous studies have established that glycogen metabolism modulates inflammatory signaling during sepsis largely through GSK3β activity, which integrates upstream signals to regulate transcriptional responses via NF-κB and CREB pathways (35, 36). In contrast, GYG1 functions at a distinct metabolic level by catalyzing the priming step of glycogen synthesis, thereby determining the cellular glycogen reserve available for rapid glycolytic activation. Our results indicate that Gyg1 depletion reduces intracellular glycogen content and dampens cytokine production without altering canonical signaling molecules such as GSK3β. Thus, GYG1 complements GSK3β-mediated immune regulation by controlling the metabolic substrate pool that sustains pro-inflammatory effector functions. This distinction highlights GYG1 as a unique upstream regulator of immunometabolic homeostasis and a potentially novel therapeutic target in sepsis.

The other four hub genes also have plausible roles in shaping immune responses. ALPL (alkaline phosphatase, tissue-nonspecific isozyme) has been implicated in detoxifying lipopolysaccharide and modulating inflammatory signaling (37). CYP1B1, a cytochrome P450 enzyme, can influence oxidative stress and lipid mediator metabolism, thereby affecting immune cell activation (38). OLAH (oleoyl-ACP hydrolase) is involved in fatty acid metabolism, and altered lipid handling has been linked to immune suppression in late-stage sepsis (39). VNN1 (vanin-1) participates in pantothenic acid metabolism and oxidative stress regulation, and its activity may influence leukocyte recruitment (40). Collectively, these genes represent interconnected metabolic nodes that can modulate both innate and adaptive immune functions during sepsis.

Our findings align with and expand current understanding of sepsis pathophysiology. Traditionally, sepsis has been conceptualized as a biphasic process, beginning with systemic hyperinflammation driven by innate immune activation, followed by a phase of immune suppression dominated by lymphocyte exhaustion. Our work highlights metabolic heterogeneity as a determinant of these immune states, with high-risk patients showing pronounced neutrophil dominance and T/NK cell suppression. Notably, our single-cell data also revealed increased platelet abundance in high-risk patients, consistent with earlier hypotheses that coagulopathy and platelet activation contribute to sepsis-related mortality. While this observation was not a primary focus of our study, it reinforces the multifaceted nature of sepsis pathobiology, in which coagulation, metabolism, and immunity are intricately interconnected.

Therapeutically, sepsis management remains largely supportive, relying on timely antibiotics, hemodynamic stabilization, and organ support. Targeted immunomodulatory therapies have had limited success, in part due to patient heterogeneity. Our immune–metabolic risk score provides a framework for patient stratification, which could inform more personalized therapeutic approaches. Recent studies have also emphasized the translational potential of biomarkers and transcriptomic risk models for patient stratification, further supporting our findings (41–44). The proof-of-concept intervention targeting GYG1 via LNP–siRNA delivery represents a novel strategy to modulate immune metabolism in sepsis. Although further preclinical optimization is required, such approaches may complement existing therapies by selectively dampening hyperactive innate immune responses without broadly suppressing immunity.

This study has several limitations. First, although we integrated bulk and single-cell transcriptomic datasets from multiple cohorts, the patient sample size for some analyses was modest, and the external validation was limited to available public datasets. Second, our *in vivo* functional validation was performed in an LPS-induced model, which recapitulates aspects of hyperinflammation but does not fully capture the complexity of clinical sepsis. Additionally, dissecting the upstream regulatory networks and detailed downstream effectors of GYG1 in immune cells may reveal broader therapeutic opportunities.

## Conclusion

In conclusion, our integrative multi-omics approach uncovered metabolic–immune heterogeneity in sepsis, established a robust immune–metabolic risk score system, and identified GYG1 as a potential metabolic driver of innate immune hyperactivation. These findings advance our understanding of the metabolic underpinnings of sepsis pathophysiology and open avenues for metabolism-targeted interventions in this complex and deadly syndrome.

## Data Availability

All the datasets analyzed in this study can be found in the Gene Expression Omnibus (GEO) at https://www.ncbi.nlm.nih.gov/geo/ with accession numbers GSE57065, GSE95233, and GSE167363.
